# Management of symptomatic uncomplicated diverticular disease (SUDD) of the colon with *Clostridium butyricum* CBM588 versus rifaximin: a retrospective cross-sectional study

**DOI:** 10.1007/s00384-025-05005-6

**Published:** 2025-10-18

**Authors:** Riccardo Urgesi, Cristiano Pagnini, Fernando De Angelis, Amjad Khan, Lorella Pallotta, Gianfranco Fanello, Pavlos Antypas, Maria Carla Di Paolo, Giuseppe Villotti, Alexander Bertuccioli, Davide Sisti, Francesco Di Pierro, Nicola Zerbinati, Maria Giovanna Graziani

**Affiliations:** 1 Gastroenterology Department, S. Giovanni Addolorata Hospital, Rome, Italy; 2 Sapienza University of Rome, Rome, Italy; 3 Department of Biochemistry, Liaquat University of Medical & Health Sciences (LUMHS), Jamshoro, Pakistan; 4 Department of Biomolecular Sciences, University of Urbino Carlo Bo, Urbino, Italy; 5 Microbiota International Clinical Society, Turin, Italy; 6 Scientific & Research Department, Velleja Research, Milan, Italy; 7 Department of Medicine and Technological Innovation, University of Insubria, Varese, Italy

**Keywords:** Symptomatic uncomplicated diverticular disease, SUDD, Probiotic, Clostridium butyricum, Rifaximin, Short chain fatty acids, SCFAs, Butyrate

## Abstract

**Background:**

Symptomatic uncomplicated diverticular disease (SUDD) is a chronic condition frequently characterized by abdominal pain, bloating, and altered bowel habits. While cyclic rifaximin is commonly used for symptom control, interest is growing in the potential role of probiotics. This study aimed to compare the clinical outcomes of patients with SUDD treated with either *Clostridium butyricum* CBM588® or cyclic rifaximin over a 12-month period.

**Methods:**

This retrospective cross-sectional study included 70 patients with a confirmed diagnosis of SUDD, treated between 2023 and 2024. Patients were divided into two groups based on treatment received: Group A (CBM588® daily for 12 months) and Group B (cyclic rifaximin 400 mg bid for 7 days per month). Clinical data, symptom profiles, and occurrence of diverticulitis were collected and compared. The primary outcome was the reduction of SUDD-related symptoms, and the secondary outcome was the incidence of acute diverticulitis episodes during follow-up.

**Results:**

A total of 56 patients completed the 12-month follow-up (31 in Group A, 25 in Group B). No significant difference was observed in the rate of symptomatic flares between groups (19.4% vs. 20%, *p* = 0.7). However, a significantly higher proportion of patients in the CBM588^®^ group reported adequate symptom relief (77.4% vs. 44%, *p* = 0.02). Improvements in bloating and tenesmus were more frequent in the CBM588^®^ group, although not statistically significant. No treatment-related adverse events were recorded.

**Conclusion:**

In this retrospective comparison, *Clostridium butyricum* CBM588^®^ demonstrated similar efficacy to rifaximin in preventing diverticulitis, with a potential advantage in subjective symptom improvement. These findings support further prospective studies to explore the role of CBM588^®^ in SUDD management.

**Trial registration**

Clinicaltrial.gov reference: NCT06852274

## Introduction

Approximately 60% of individuals over the age of 60 living in industrialized countries will develop colonic diverticula [1]. Among these, symptomatic disease is likely to occur in 10–25% of cases [2]. Complications of diverticulitis can be serious and potentially life-threatening, including bowel perforation, abscess, fistula, bleeding, and strictures leading to obstruction. Surgical intervention may become necessary, ranging from endoscopic or percutaneous procedures to laparoscopic or open surgery. In the USA alone, the number of hospitalizations for patients with a primary discharge diagnosis of diverticular disease (diverticulosis and diverticulitis) increased by 32% from 220,896 to 293,530, and complications of diverticulitis account for approximately 130,000 hospitalizations annually [3, 4].

A substantial proportion of patients who experiences an episode of acute diverticulitis continue to suffer from recurrent abdominal pain, altered bowel habits, and bloating in the absence of overt inflammation. This specific subgroup is now recognized as suffering from symptomatic uncomplicated diverticular disease (SUDD) [5, 6]. According to the International Consensus on Diverticulosis and Diverticular Disease, SUDD is defined as a chronic inflammatory condition characterized by persistent gastrointestinal symptoms, elevated systemic inflammatory markers, increased mucosal cytokine expression, and chronic inflammatory infiltrates in colonic tissue [6]. Several clinical studies have reported symptomatic improvement in these patients using 5-aminosalicylic acid or treatments that modulate bacterial–mucosal interactions, such as rifaximin and probiotics [7–12]. Patients with SUDD present with a broad spectrum of symptoms, and effective preventive strategies could significantly reduce both symptom burden and disease-related morbidity.

Short-chain fatty acids (SCFAs) play a pivotal role in maintaining intestinal homeostasis, and their deficiency has been implicated in the pathogenesis of several inflammatory and metabolic disorders. Among SCFAs, butyrate is considered particularly crucial for colonic health due to its trophic effects on colonocytes, anti-inflammatory properties, and its role in enhancing mucosal barrier integrity and mucus production [13]. Despite its benefits, oral administration of butyrate is limited by formulation challenges, including the need for high doses and difficulties in delivering the active compound effectively to the colon.

*Clostridium butyricum* is a butyrate-producing human gut symbiont that has demonstrated clinical benefits in a range of gastrointestinal and systemic conditions, including intestinal infections, mucosal injury, irritable bowel syndrome (IBS), inflammatory bowel disease, neurodegenerative diseases, metabolic disorders, and colorectal cancer [14]. Notably, it is the only probiotic currently available in Europe with confirmed butyrate-producing capacity.

Given the similarities between SUDD and irritable bowel syndrome (IBS)—including abnormal colonic motility, visceral hypersensitivity, low-grade inflammation, and neuropeptide alterations [15]—and the known efficacy of butyrate in IBS, we hypothesized that *Clostridium butyricum* CBM588^®^ could provide protective and symptomatic benefits in diverticular disease.

The aim of this retrospective, cross-sectional study was to evaluate the preventive effect of *Clostridium butyricum* CBM588^®^ (Butirrisan^®^) compared with cyclic rifaximin therapy in patients with colonic diverticulosis in a real-life single-center population. The primary outcomes assessed were the incidence of acute diverticulitis and the persistence of gastrointestinal symptoms during a 12-month follow-up period.

## Materials and methods

### Study design and patient population

This was a retrospective, observational, cross-sectional study based on the review of clinical records from an outpatient gastroenterology clinic. Patients with a diagnosis of symptomatic uncomplicated diverticular disease (SUDD) who attended the clinic between January 2023 and January 2024 were screened for inclusion. Eligible patients were followed for a 12-month period after the initial visit.

Inclusion criteria consisted of the following: age ≥ 18 years, documented colonic diverticulosis confirmed by colonoscopy or abdominal CT, and at least one previous episode of mild to moderate diverticulitis requiring medical treatment. Exclusion criteria included the following: history of inflammatory bowel disease (IBD), gastrointestinal or abdominal malignancy, prior abdominal surgery, severe comorbidities, pregnancy or breastfeeding, use of antibiotics or probiotics within 4 weeks before the baseline visit, and ongoing abdominal symptoms at the time of enrollment.

### Study groups and interventions

A total of 75 patients were initially identified. After applying the exclusion criteria and accounting for early dropouts, 70 patients were included in the final analysis. Based on clinical record review, patients were assigned to one of two treatment groups according to their prescribed management:

- Butirrisan^®^ group (*n* = 35): Patients received *Clostridium butyricum* CBM588^®^ (Butirrisan^®^, PharmExtracta^®^ S.p.A., Pontenure, Italy) at a dose of three 30 mg tablets daily (≥ 4.5 × 10^5^ CFU/tablet), administered continuously for the first month and then for 14 days per month over the subsequent 11 months. All patients received dietary fiber supplementation (a general advice to consume about 15–20 g fiber/day).
- Rifaximin group (*n* = 35): Patients received rifaximin 400 mg twice daily for 7–10 days each month for 12 months, in addition to fiber supplementation (a general advice to assume about 15–20 g fiber/day).

Treatment allocation was not randomized but determined retrospectively from the clinical management documented in patient records. The actual choice of the treatment was exclusively decided by the physician, who prescribed based on his experience and knowledge, and discussed with the patients the possible options. The patients were then given a prescription and they could get the drug or the probiotic directly in the Pharmacy store. At the time of the first visit, patients were advised to return to the clinic if they experienced abdominal pain, fever, or other symptoms suggestive of diverticulitis. Acute diverticulitis was defined by the presence of left lower quadrant abdominal pain and tenderness, fever > 38 °C, or leukocytosis (WBC > 10,000/mm^3^). Symptoms’ flare was defined by the onset of abdominal symptoms (pain, discomfort, alteration of bowel habit) requiring the seek for medical consultation (general physician, gastroenterologist) besides the regular scheduled visits.

### Follow-up and dropouts

The study concluded with a 12-month follow-up assessment. Of the 70 patients included, 56 (80%) completed the follow-up: 31 in the Butirrisan^®^ group (88.6%) and 25 in the rifaximin group (71.4%). A total of 14 patients were excluded from the final analysis: 7 were lost to follow-up (4 in the treatment group and 3 in the control group), and 7 discontinued the study due to intercurrent conditions requiring systemic antibiotic treatment (e.g., *H. pylori* eradication, respiratory and urinary tract infections).

### Outcomes and data collection

The primary outcome was the clinical effectiveness of *Clostridium butyricum* CBM588^®^ versus rifaximin in reducing SUDD-related symptoms over the 12-month follow-up. Symptom assessment included the following:

- A binary patient-reported outcome adapted from Krokowicz et al. [16]: “Did you observe adequate relief of diverticulosis-related abdominal pain or discomfort during the past 12 months of treatment?” (Yes/No)
- A symptom-specific questionnaire adapted from Lahat et al. [17], consisting of nine items evaluating frequency, duration, and severity of: abdominal pain, bloating, tenesmus, altered bowel habits, impact on daily activities, mood disturbances, and desire for treatment. The original English questionnaire was translated into Italian for patient use.

The secondary outcomes were the incidence of acute diverticulitis episodes during follow-up and the need for surgical or additional pharmacological intervention.

The study was approved by Local Ethical Committee and registered in Clinicaltrial.gov web site (NCT06852274).

### Statistical analysis

Descriptive statistics were used to summarize baseline characteristics and outcomes. Categorical variables were compared using Pearson’s chi-squared test. A *p*-value of < 0.05 was considered statistically significant. All analyses were performed using statistical software MedCalc version 12.5.

## Results

After 12 months of follow-up, 56 of the 70 participants (80%) completed the study and were included in the final analysis. Baseline demographic characteristics are summarized in Table 1. The mean age in the study group (*n* = 31) was 66.3 years (± 12.5), compared to 63.4 years (± 12.6) in the control group (*n* = 25). There was no statistically significant difference in sex distribution between groups; in the study group, 16 of 31 participants (51.6%) were male. The average age at the first episode of diverticular disease was 53.17 years (± 13.64) in both groups.

**Table 1:** Patient baseline characteristics

*n*	56
Sex (male) (%)	23 (44.6)
Age at first AD attack [mean (SD)]	53.17 (13.64)
No. of past AD attacks [mean (SD)]	2.08 (1.64)
Time since last documented AD attack (months) (SD)	7.8 (2.9)
DICA Endoscopic Classification* *n* (%)	
1	23 (41.1)
2	25 (44.6)
3	8 (14.3)
Comorbidities* n *(%)	
Cardiovascular disease	31 (55.3)
Endocrine disorders	6 (10.7)
No comorbidities	19 (33.9)
Concomitant medications *n *(%)	
Aspirin/NSAIDs	5 (8.9)
Other**	38 (67.9)
None	13 (23.2)
Previous abdominal operations*** *n* (%)	23 (41.1)

*DICA score is the sum of different parameter: the extension of Colonic Diverticulosis, the number of Diverticula per region, the presence and the type of inflammation, the presence and the type of possible complications

**Other medications: thyroid replacement hormones, beta-blockers, statins, calcium channel blockers, proton pump inhibitors, oral diabetic treatment.

***Previous abdominal operations: uterus and annexes resection, appendectomy, cholecystectomy

Treatment with cyclic rifaximin and *Clostridium butyricum* CBM588^®^ was well tolerated, with no reported adverse events.

During the 12-month follow-up, the occurrence of symptomatic flare was observed in 6 out of 31 (19.4%) in the CBM588^®^ group and in 5 out of 25 (20%) in the control group, with no statistically significant difference between groups (*p* = 0.7).

Regarding clinical symptomatology, abdominal pain occurred less than once per week in 74.2% of patients in the CBM588^®^ group. The duration of abdominal pain episodes was under one hour in 29 of 31 patients (93.55%), and the pain was rated as absent or mild in 28 of 31 patients (90.3%) (Table 2). Even though quite variable results have been observed for some aspects, a general reduction in both the severity and frequency of symptoms was observed in the treatment group. In particular, abdominal bloating and tenesmus improved more frequently in the CBM588^®^ group compared to controls (19.3% vs. 8%), although the difference did not reach statistical significance, likely due to the small sample size.

**Table 2:** Patients response to questionnaire at baseline (all the patients) and after 12 months of follow-up (control vs. study group)

	Start of the study	Control group (rifaximin)	Study group (*C. butyricum* CBM588®)
*n *	56	25	31
Age [mean (SD)]	64.01±12.2	66.4±12.6	66.3±12.5
Sex (male) (%)	23 (41.1)	7 (28)	16 (51.6)
Disease activity according to PGA (%)			
remission	18 (32.1)	7 (28)	13 (41.9)
active disease	33 (58.9)	16 (64)	15 (48.4)
Moderate disease activity	5 (8.9)	2 (8)	3 (9.7)
Abdominal pain, frequency (%)			
1 Less than once a week	21 (37.5)	9 (36)	23 (74.2) *
2 1–2 times a week	25 (44.6)	8 (32)	4 (12.9)
3 3–6 times per week	8 (14.3)	5 (20)	2 (6.4)
4 Daily	2 (3.6)	3 (12)	2 (6.4)
Abdominal pain, duration (%)			
1 <30 min	15 (26.8)	5 (20)	13 (41.9)
2 0.5 h to 1 h	35 (62.5)	11 (44)	16 (51.6)
3 1–6 h	5 (8.9)	9 (36)	2 (6.5) *
4 >6 h	1 (1.8)	0	0
Abdominal pain, severity (%)			
0 No pain	5 (8.9)	5 (20)	15 (48.4) *
1 Mild pain	16 (28.6)	7 (28)	13 (41.9)
2 Moderate pain	32 (57.1)	10 (40)	3 (9.7) *
3 Severe pain	2 (3.6)	3 (12)	0 *
4 Very severe pain	1 (1.8)	0	0
Number of additional symptoms (%) (choose any of the following: bloating, tenesmus, change in bowel habits)			
0	3 (5.4)	2 (8)	6 (19.3)
1	25 (44.6)	6 (24)	8 (25.8)
2	21 (37.5)	2 (8)	2 (6.5)
3	7 (12.5)	15 (60)	15 (48.4)
Missed activities during last 2 weeks (%)	12 (21.4)	0	0
Woken up at night during last 2 weeks (%)	33 (58.9)	4 (16)	1 (3.2)
Experienced lack of energy in last 2 weeks (%)	42 (75)	9 (36)	8 (25.8)
Felt anxious or depressed in last 2 weeks (%)	15 (26.8)	2 (8)	5 (16.1)
Felt the need to change treatment in last 2 weeks (%)	18 (32.1)	0	2 (6.5)

*p<0.05 in study vs. control group

No episodes of acute diverticulitis were observed in either group during the study period.

**Fig. 1:**
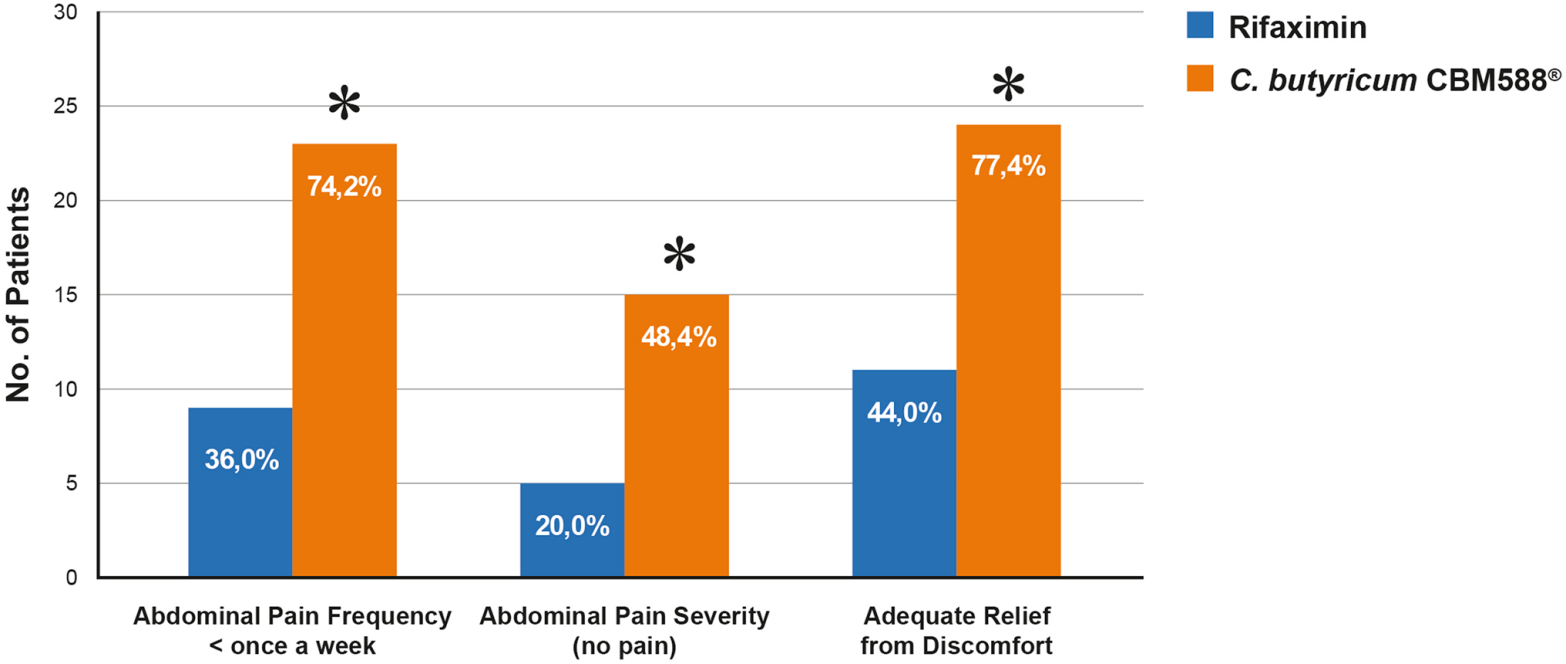
Significant improvement of symptoms in *C. butyricum* CBM588^®^ vs. control group. *p < 0.05

Subjective symptom improvement, assessed by a single yes/no question (“Did you experience adequate relief from diverticulosis-related abdominal pain or discomfort within the past 12 months?”), was significantly higher in the treatment vs. control group (77.4% vs. 44% *p* = 0.02). Globally, patients in the CBM588^®^ group showed significant symptoms amelioration comparing with control group, for 74.2% vs. 36% referred abdominal pain frequency less than once a week, 48.4% vs. 20% referred no pain at all, and 77.4% vs. 44% reported adequate relief of abdominal discomfort (*p* < 0.05, Fig. 1).

In particular, the most notable improvement in perceived adequate relief occurred in the DICA 1 subgroup of the CBM588^®^ group (Tables 3).

**Table 3:** Adequate relief to discomfort after treatment at the the end of follow-up per DICA groups

DICA	n (%)	Control group	Study group
1	23 (41.1)	12 (48)	11 (35.5)
2	25 (44.6)	8 (32)	17 (54.8)
3	8 (14.3)	5 (20)	3 (9.7)
Adequate relief of diverticulosis related discomfort		11/25 (44)	24/31 (77.4)*
DICA 1		6/12 (50)	10/11 (90.9)*
DICA 2		4/8 (50)	13/17 (76.5)
DICA 3		1/5 (20)	1/3 (33.3)

*p<0.05

## Discussion

Current clinical guidelines emphasize that treatment of symptomatic uncomplicated diverticular disease (SUDD) focuses primarily on symptom relief and prevention of complications, mainly acute diverticulitis. Various therapeutic approaches have been proposed, including bulking agents, spasmolytics, topical antibiotics, and anti-inflammatory drugs, each targeting different potential pathophysiological mechanisms [18–20]. However, the efficacy of some treatments remains controversial. For instance, the routine use of antibiotics in SUDD is debated. Nevertheless, rifaximin, despite an unclear mechanism of action in this context, has demonstrated effectiveness in symptom management in several studies [21–23]. De Bastiani et al. [24], in a real-life cohort, confirmed rifaximin’s efficacy by showing significant reduction in VAS scores across multiple symptoms.

Butyrate, a key short-chain fatty acid (SCFA), plays a critical role in maintaining gut homeostasis and modulating inflammation. Its deficiency has been implicated in various gastrointestinal and systemic diseases, including inflammatory bowel disease (IBD), colorectal cancer, and cardiometabolic disorders. SCFAs, including acetate, propionate, and butyrate, are produced by specific gut microbiota taxa, whose abundance can be influenced by diet, prebiotics, and probiotics [25–27].

Butyrate promotes intestinal barrier integrity by upregulating tight junction proteins (claudin-1, zonula occludens-1, and occludin) [28], enhancing mucus production via Mucin 2 expression [29], and reducing oxidative stress by protecting against H2O2-induced DNA damage. In IBD patients, a reduction of butyrate-producing bacteria such as *Roseburia* spp. and *Faecalibacterium prausnitzii* correlates with decreased SCFA levels and impaired anti-inflammatory effects [30, 31]. Butyrate and propionate further modulate immune responses by stimulating T-regulatory cell proliferation and inhibiting pro-inflammatory pathways, including NF-κB, via GPR43 and HDAC inhibition [32–40].

Oral butyrate supplementation has been explored as adjunct therapy in IBD, showing some benefits in reducing clinical scores and inflammatory markers [41], although larger randomized studies have yielded mixed results [42]. Similarly, SCFA enemas have been trialed for radiation proctitis with limited success [43, 44].

In the present study, we investigated the efficacy of *Clostridium butyricum* CBM588^®^, a butyrate-producing probiotic, in reducing symptomatic diverticulitis occurrence and improving clinical symptoms in SUDD patients. This was achieved through a retrospective cross-sectional investigation evaluating symptom frequency, severity, and patient-reported outcomes over 12 months, following the framework established by Krokowicz et al. [16].

SUDD is a chronic, recurrent condition affecting quality of life and physical activity, with up to 80% recurrence rate of abdominal symptoms within 5 years after nonsurgical treatment [20]. Various non-pharmacologic strategies, such as dietary fiber modification to increase stool bulk and reduce intraluminal pressure, have been proposed [45].

Pharmacological interventions, including rifaximin, have demonstrated symptom reduction compared to fiber alone, supporting their use in SUDD management [7, 24], and anti-inflammatory agents, such as mesalazine, also contribute to symptom control and relapse prevention [8, 9, 46, 47]. Rifaximin is thought to reduce bacterial overgrowth within diverticula, decreasing mucosal inflammation and symptoms related to dysbiosis [48]. Its cyclical administration improves symptoms and quality of life with a good safety profile, as supported by several observational and controlled studies [23, 49]. Guidelines recommend scheduled cyclical treatment over on-demand therapy for optimal symptom control and complication prevention [22, 50–52]. Although progression from SUDD to acute diverticulitis is relatively infrequent (estimated around 3%) [53], rifaximin combined with fiber may modestly reduce this risk [7, 11, 54]. Nonetheless, the use of antibiotics for primary prevention of diverticulitis in asymptomatic diverticulosis lacks robust therapeutic justification and is not recommended.

Our findings showed symptomatic improvement and a trend toward reduced symptom severity in the CBM588^®^ group, with particular benefit regarding abdominal bloating and tenesmus, which are impactful on quality of life. These effects likely stem from butyrate’s roles in energy metabolism of colonocytes, modulation of intestinal permeability, oxidative stress reduction, and mucosal healing [26, 55].

The study supports the potential for CBM588^®^ as part of a multimodal preventive strategy, alone or combined with other agents, particularly for patients at high risk of recurrence.

## Conclusion

In conclusion, the results of this study suggest that supplementation with *Clostridium butyricum* CBM588^®^ may represent an effective and safe option for managing symptoms in patients with symptomatic uncomplicated diverticular disease (SUDD). The probiotic showed potential benefits in reducing both the frequency and severity of symptoms, thereby improving patients’ quality of life. Although the protective effect against diverticulitis recurrence appears promising, further confirmation through larger studies with extended follow-up is warranted.

The action of *C. butyricum*, through butyrate production, contributes to modulating local inflammation, strengthening the intestinal barrier, and promoting colonic mucosal health, confirming the crucial role of the gut microbiota and its metabolites in the pathophysiology of diverticular disease.

These findings pave the way for future therapeutic strategies focused on targeted modulation of the gut microbiota and the use of butyrate-producing probiotics, potentially in combination with conventional therapies, to improve symptom control and prevent complications.

## Data Availability

Data available on request.
